# Controlled bioreduction of silver ions to nanosized particles on a porous magnetic-biopolymer of carboxymethyl cellulose, Fe_3_O_4_/CMC-Ag NPs, serving as a sustainable nanocatalyst

**DOI:** 10.1039/d4na00866a

**Published:** 2024-12-11

**Authors:** Mojtaba Azizi, Mahdi Jafari, Sadegh Rostamnia

**Affiliations:** a Organic and Nano Group (ONG), Department of Chemistry, Iran University of Science and Technology (IUST) PO Box 16846-13114 Tehran Iran rostamnia@iust.ac.ir srostamnia@gmail.com

## Abstract

A magnetic-biopolymer composite of carboxymethyl cellulose (CMC), designated as Fe_3_O_4_@CMC, was synthesized featuring remarkable stability and an active surface with a green biosynthetic method. This composite was engineered to serve as a substrate for stabilizing silver nanoparticles (Ag NPs) with enhanced functional properties. The catalytic efficacy of the nanocatalyst, incorporating Ag NPs at concentrations of 3%, 7%, and 10%, was evaluated for the reduction of the toxic compound 4-nitrophenol to the beneficial 4-aminophenol. Among the tested configurations, the formulation containing 10% silver nanoparticles, in conjunction with Euphorbia plant extract as a bioreducing agent, exhibited the highest reduction efficiency and favorable reaction kinetics, rendering it the optimal choice. The apparent rate constant (*K*_app_) was assessed by fine-tuning the catalyst parameters, while the reaction mechanism was further elucidated by adjusting the concentrations of NaBH_4_ and 4-nitrophenol. Notably, the catalyst demonstrated good stability over five consecutive reduction cycles and could be easily retrieved from the reaction mixture using an external magnet.

## Abstract 2

A magnetic-biopolymer composite of carboxymethyl cellulose (CMC), designated as Fe_3_O_4_@CMC, was synthesized featuring remarkable stability and an active surface with a green biosynthetic method.

## Graphical abstract

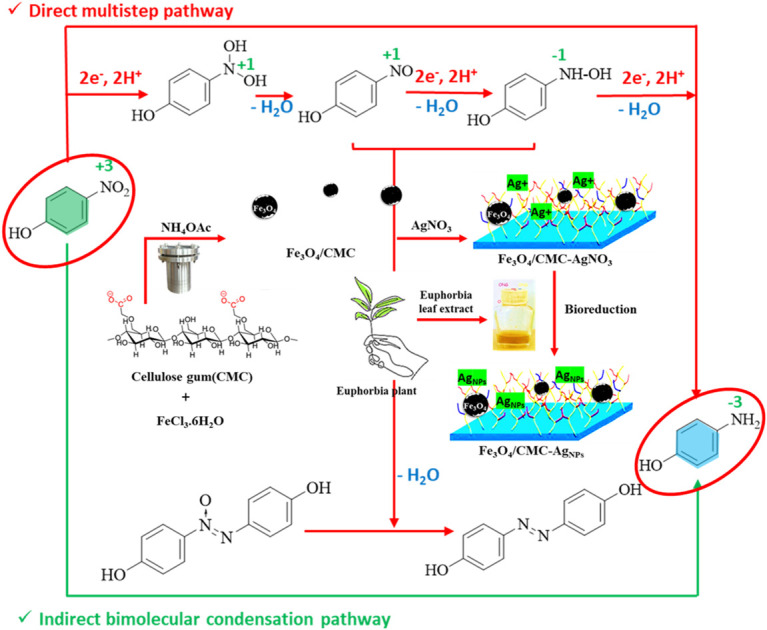

## 1 Introduction

4-Nitrophenol is a nitroaromatic pollutant utilized as an intermediate in various industries, including dyes, pharmaceuticals, polymers, insecticides, and agriculture. Its presence poses risks to human and animal cells as well as natural ecosystems. To address this environmental issue, numerous methods such as absorption, ion exchange, electrochemical techniques, and catalytic reduction have been employed, with catalytic reduction being particularly effective due to its cost-efficiency and high efficacy.^1^ This method often involves the use of metal nanoparticles such as AuNPs, AgNPs, PdNPs, PtNPs, Co-NiNPs, CeO_2_NPs, NiNPs, and CuNPs in the presence of NaBH_4_ to convert 4-nitrophenol (4-NP) into 4-aminophenol (4-AP), a valuable, non-toxic product used in pharmaceuticals, dyes, and lubricants.^2–8^ Thus, developing eco-friendly technologies for treating such compounds in aquatic environments is essential.^9^

Silver nanoparticles (Ag NPs) are recognized for their advantageous properties, including affordability, varied crystal facets, tunable plasmon resonance, and a high surface-to-volume ratio. They exhibit diverse functions such as catalysis,^17–19^ dye degradation,^3^ photocatalysis,^20^ antibacterial activity,^21^ anti-cancer properties,^22,23^ biological applications,^24^ electrochemical uses,^25^ and drug delivery.^26^ However, challenges arise from difficulties in recovery, comparatively larger particle sizes, the formation of surface oxide layers, and self-aggregation, which diminish the active surface area and catalytic efficacy. Consequently, efforts have been directed towards enhancing environmental compatibility,^27^ controlling size,^28^ improving substrate stability,^18,29^ employing green synthesis methods,^19^ boosting performance,^30^ increasing accessibility,^31^ and conducting optimization studies. The utilization of robust substrates for the stabilization of nanoparticles constitutes a viable approach to addressing these challenges.

Magnetic Fe_3_O_4_ nanoparticles demonstrate remarkable magnetic and field-dependent optical properties, rendering them suitable for diverse applications such as catalysis, removal of heavy metal ions,^10^ dye removal,^11^ biodiesel,^12^ electrocatalysis,^13^ and drug delivery.^14,15^ Additionally, they function as substrates for material growth, aiding in separation processes. However, unmodified nanoparticles are prone to self-aggregation due to oxidation from air and moisture exposure, coupled with their inherent magnetism, which adversely affects their uniformity. To mitigate this challenge, nanoparticle surfaces are frequently coated with polymers or surfactants to diminish magnetic interactions and regulate their magnetic behavior, thereby inhibiting surface reactions.^15,16^

**Scheme 1:**
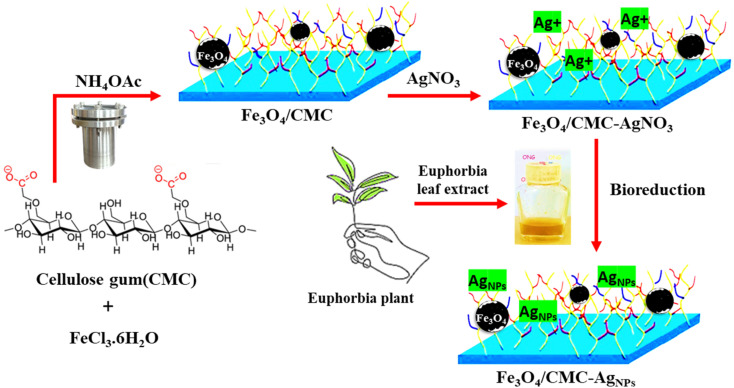
Preparation of biopolymer-magnetic Fe_3_O_4_/CMC-AgNP nanocomposite.

This study explores the integration of carboxymethyl cellulose (CMC), a biopolymer, with Fe_3_O_4_ magnetic nanoparticles to serve as a substrate for regulating the growth of AgNPs, as illustrated in Scheme 1. The structural identity of the Fe_3_O_4_/CMC-AgNP nanocomposite was confirmed through structural determination analyses. Additionally, UV-visible absorption spectroscopy was employed to achieve optimal catalytic activity in minimal time and to evaluate key optimization parameters, including the dosage of the catalyst and its recyclability. Ultimately, this research highlights the advancement of eco-friendly biopolymer-based catalysts, proposing a solution to mitigate environmental pollution while demonstrating significant effectiveness.

## 2 Experimental

### 2.1 Materials

All chemicals were utilized in their received state without any additional purification processes. Sodium carboxymethyl cellulose (average molecular weight = 90.00 kg mol^−1^) was obtained from Sigma Aldrich, serving as a source of natural polymers. Iron(iii) chloride hexahydrate (FeCl_3_·6H_2_O) from Merck was employed as a precursor for the synthesis of iron oxide nanoparticles (Fe_3_O_4_). Silver nitrate (from Merck) was used as a precursor for the fabrication of silver nanoparticles (Ag-NPs).

### 2.2 Preparation of Fe_3_O_4_/CMC

The composite of magnetic nanoparticles, coated with the biopolymer carboxymethyl cellulose (CMC), was successfully synthesized using an *in situ* solvothermal method. Initially, ammonium acetate (0.385 g, 5 mmol) was incorporated into a solution composed of ethylene glycol (7 mL) and iron(iii) chloride hexahydrate (135 mg, 0.5 mM), while being continuously stirred. Subsequently, CMC (*M*_w_ = 90.0 kg mol^−1^ and purity = 99.5%) was added to the resulting brown mixture, which was stirred vigorously for 45 minutes. The homogeneous solution was then transferred to a stainless-steel autoclave lined with Teflon and subjected to heating at 200 °C for 12 hours. Following the cooling process, the resultant precipitate was isolated using an external magnetic field and thoroughly washed multiple times with deionized water and ethanol. Finally, the dark Fe_3_O_4_/CMC precipitate was dried at a temperature of 60 °C.

### 2.3 Preparation of the Euphorbia plant extract

The aerial parts at the flowering stage were thoroughly dried and subsequently ground to produce an extract from the Euphorbia plant. The resulting powder was then immersed in ethanol, and the solution was subjected to filtration three times following a 36 hour period.

### 2.4 Preparation of Fe_3_O_4_/CMC-AgNPs

A total of 0.1 g of Fe_3_O_4_/CMC was suspended in 25 milliliters of deionized water. Subsequently, 100 ml of a silver nitrate solution, with a concentration of 10 mg L^−1^, was introduced into the mixture and agitated for a period of 30 min. Following this, the Euphorbia plant extract solution was added gradually, and the mixture was stirred for 8 h at room temperature. The resulting magnetic precipitate was then rinsed with deionized water and ethanol and subsequently dried at a temperature of 60 °C.

### 2.5 Reduction of 4-nitrophenol using the Fe_3_O_4_/CMC-AgNP nanocomposite

To evaluate the catalytic activity of the synthesized nanocatalyst, a solution containing 0.072 mM of 4-nitrophenol was prepared, to which 10 mg of NaBH_4_ was added to generate nitrophenolate ions. Subsequently, 8 mg of the catalyst was introduced, and the catalytic reduction process was monitored using a UV-vis spectrophotometer over a wavelength range of 200–500 nm at various time intervals. The reusability of the Fe_3_O_4_/CMC-AgNP nanocatalyst was also assessed; it was magnetically separated from the reaction mixture post-use, washed with ethanol and distilled water, and dried for future applications. The catalytic reduction efficiency of 4-NP to 4-AP was calculated using formula (1):1
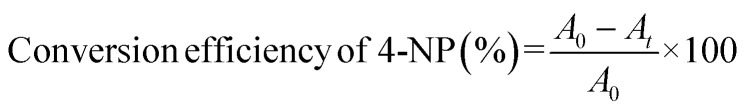
*A*_0_ is the initial absorption of nitrophenolate anions and *A*_*t*_ is the absorption in different time intervals (*t*).

## 3 Results and discussion

The Fe_3_O_4_/CMC-AgNP composites were synthesized according to the method previously reported by our own group through an *in situ* modification strategy (Scheme 1). In summary, CMC was included among the components of magnetic Fe_3_O_4_ and CMC-modified Fe_3_O_4_ was synthesized in one step using the solvothermal method. The presence of the CMC shell on the Fe_3_O_4_ core, in addition to preventing the reduction of magnetic energy due to agglomeration, also contributes to the next step of better stabilizing silver nanoparticles. In conclusion, the process of immobilizing silver nanoparticles (AgNPs) onto magnetic carboxymethyl cellulose, facilitated by the extract of the Euphorbia plant, has been identified as a sustainable and environmentally friendly method for the reduction of Ag ions.

**Fig. 1:**
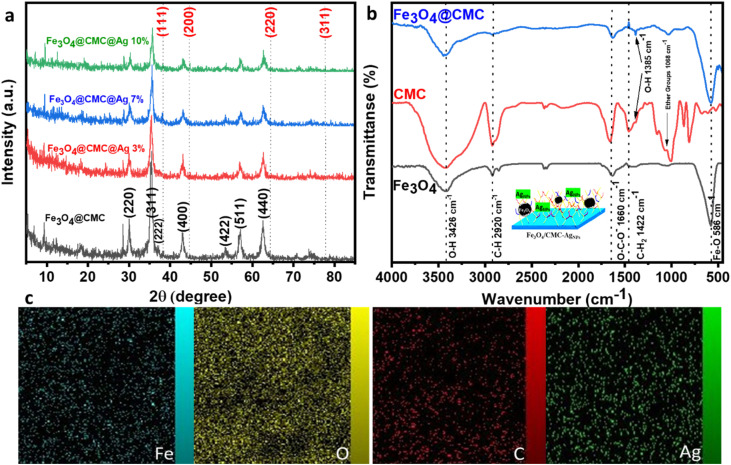
(a) XRD patterns of Fe_3_O_4_/CMC, Fe_3_O_4_/CMC-Ag-3%, Fe_3_O_4_/CMC-Ag-7% and Fe_3_O_4_/CMC-Ag-10%. (b) FT-IR spectra of Fe_3_O_4_, CMC and Fe_3_O_4_/CMC. (c and d) EDX mapping image of Fe_3_O_4_/CMC-Ag-7%.

X-ray diffraction (XRD) analysis was employed to examine the crystal structure of Fe_3_O_4_, Fe_3_O_4_/CMC and the Fe_3_O_4_/CMC-AgNP catalyst (Fig. 1a). The XRD pattern of pure Fe_3_O_4_/CMC reveals eight peak indices corresponding to the synthetic pattern of Fe_3_O_4_, specifically at 30° (220), 35.47° (311), 37.23° (222), 42.93° (400), 53.5° (422), 57.11° (511), and 62.53° (440), signifying the high purity of Fe_3_O_4_ nanoparticles. Notably, CMC peaks are absent due to the amorphous nature of the broad peak at 22°. The presence of Fe_3_O_4_ peaks confirms the successful synthesis of the composite. Additionally, the diffraction pattern for the Fe_3_O_4_@CMC@AgNP nanocomposite, which incorporates varying percentages of Ag nanoparticles, displays peaks at 38.25° (111), 44.86° (200), 64.37° (220), and 77.76° (311). An increase in the Ag percentage is associated with enhanced intensity of the Ag peak indicators. The fundamental pattern of Fe_3_O_4_ remains intact in the presence of Ag nanoparticles, though a slight reduction in peak intensity suggests partial structural degradation; overall, composites with differing Ag percentages have been effectively synthesized.

The FT-IR spectrum of pristine Fe_3_O_4_ and Fe_3_O_4_/CMC, as depicted in Fig. 1b, reveals several key vibrational peaks. The Fe_3_O_4_ pattern demonstrates a peak at 586 cm^−1^ associated with Fe–O vibrations, 1577 cm^−1^ corresponding to O–H bending vibrations, and 3426 cm^−1^ linked to O–H stretching, all of which are also observed in the Fe_3_O_4_/CMC composite. In the CMC spectrum, a peak at 2920 cm^−1^ indicates stretching vibrations, while the COO-carboxylate band at 1613 cm^−1^ represents asymmetric stretching. Additional peaks at 1422 cm^−1^ pertain to C–H stretching, 1077 cm^−1^ is related to ether groups on CMC surfaces, and 1384 cm^−1^ corresponds to O–H bending. This composition of the nanocatalyst is effectively illustrated in the composite and exhibits stability.^21^

**Fig. 2:**
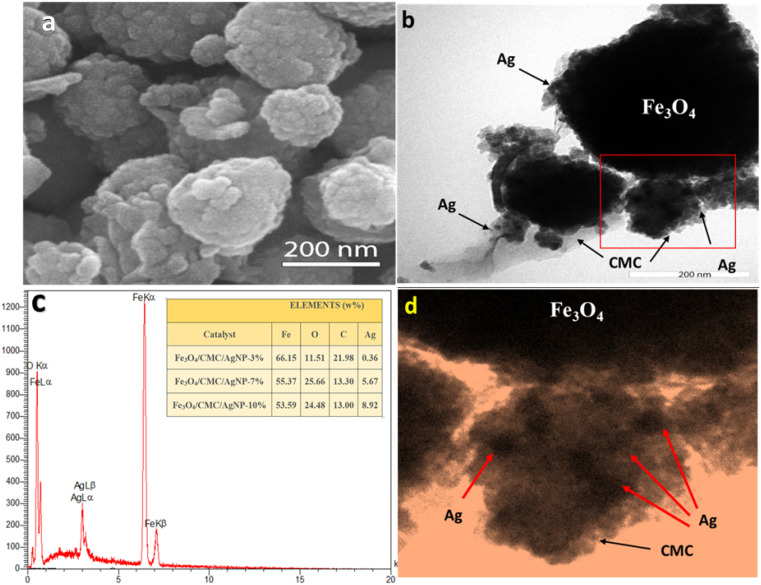
(a) SEM image of Fe_3_O_4_/CMC-Ag-7%. (b and d) TEM image of Fe_3_O_4_/CMC-Ag-7%. (c) EDX spectrum of Fe_3_O_4_/CMC-Ag-7%.

A scanning electron microscopy (SEM) analysis was conducted to evaluate the morphology and surface characteristics of the nanocatalyst. The observed magnetic Fe_3_O_4_ nanoparticles exhibited a spherical shape, averaging 300 nm in size with a uniform dispersion. This irregular surface enhances the adsorption of 4-NP molecules (Fig. 2a). Transmission Electron Microscopy (TEM) revealed a composite core–shell structure featuring Fe_3_O_4_ and CMC, displaying a low-contrast substrate with uniformly dispersed silver nanoparticles, thereby preventing their aggregation (Fig. 2b). Energy Dispersive Spectroscopy (EDS) mapping confirmed the uniform distribution of C, O, Fe, and Ag elements (Fig. 1c). EDS analysis indicated elements percentage (Fig. 2c and d). The data showed that the iron content decreased with increasing Ag percentages and Euphorbia plant reduction, resulting in iron proportions of 66.15%, 55.37%, and 53.6% for composites with 3%, 7%, and 10% Ag, respectively. After washing, the Ag percentage diminished, indicating the nanoparticles' confinement within structural cavities. The final composite percentages for Ag nanoparticles were recorded as 0.36%, 5.67%, and 8.92%, reflecting the effective synthesis and dispersion of the nanoparticles with minimal hysteresis.

**Fig. 3:**
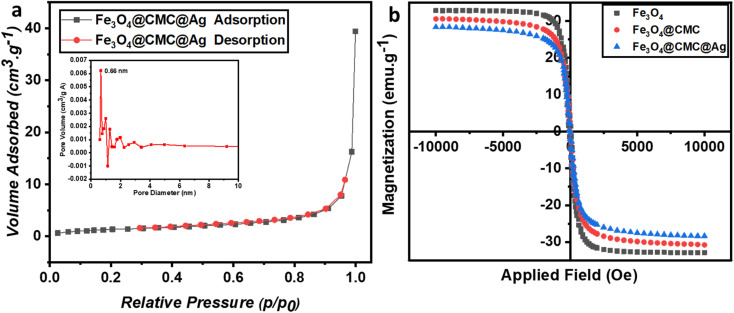
(a) N_2_ adsorption–desorption isotherms and pore diameter of Fe_3_O_4_/CMC-Ag-7%. (b) Magnetic hysteresis loops for pristine Fe_3_O_4_, Fe_3_O_4_/CMC, and Fe_3_O_4_/CMC-Ag-7%.

To examine properties such as specific surface area, volume, and pore size, N_2_ physical adsorption analysis was conducted on the Fe_3_O_4_/CMC-AgNP composite (Fig. 3a). The adsorption–desorption isotherm displayed a type IV characteristic, indicating a mesoporous structure. The specific active surface area of Fe_3_O_4_/CMC-AgNPs was measured to be 4.95 m^2^ g^−1^, significantly lower than that of pristine Fe_3_O_4_ reported in other studies, suggesting that the cavities of Fe_3_O_4_ were completely filled during the composite synthesis. The average pore diameter was approximately 27 nm, with a pore volume of 0.66 cm^3^ g^−1^. Consequently, the arrangement of composite components within the pores and on the magnetic substrate was validated.

To evaluate the magnetic properties, which are the defining characteristic of the synthesized composite and facilitate its separation after each use, VSM analysis was conducted (Fig. 3b). The magnetic saturation (Ms) values for Fe_3_O_4_, Fe_3_O_4_/CMC, and Fe_3_O_4_/CMC-AgNPs were found to be 32.82 emu g^−1^, 30.54 emu g^−1^, and 28.35 emu g^−1^, respectively. The incorporation of the non-magnetic components CMC and AgNPs leads to a reduction in magnetic strength compared to pure Fe_3_O_4_. Nonetheless, these composites retain sufficient magnetic properties for reusability and effective separation in solution, as well as for the reduction of nitroaromatic compounds.

The catalytic efficacy of the Fe_3_O_4_/CMC-AgNP nanocomposite was evaluated in the reduction of the nitroaromatic compound 4-nitrophenol (4-NP) using sodium borohydride (NaBH_4_) as the reducing agent. Ultraviolet-visible (UV-vis) spectroscopy was utilized to monitor the reaction progression. Initially, 4-NP exhibited an absorption peak at 317 nm. Following the introduction of NaBH_4_, a color transition from light yellow to dark yellow occurred, indicating the transformation of 4-NP to 4-nitrophenolate, which corresponded to an enhanced absorption peak around 400 nm linked to the nitrophenolate product. Moreover, the kinetics of the reduction of 4-NP to 4-AP in the presence of NaBH_4_ were also analyzed to further elucidate the catalyst's performance. According to formula (2):2
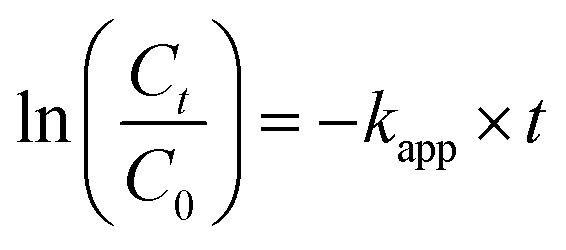


In this context, *C*_0_ denotes the initial concentration of 4-NP, *C*_*t*_ signifies the concentration of 4-NP at time *t*, and *k*_app_ refers to the apparent rate constant.

**Fig. 4:**
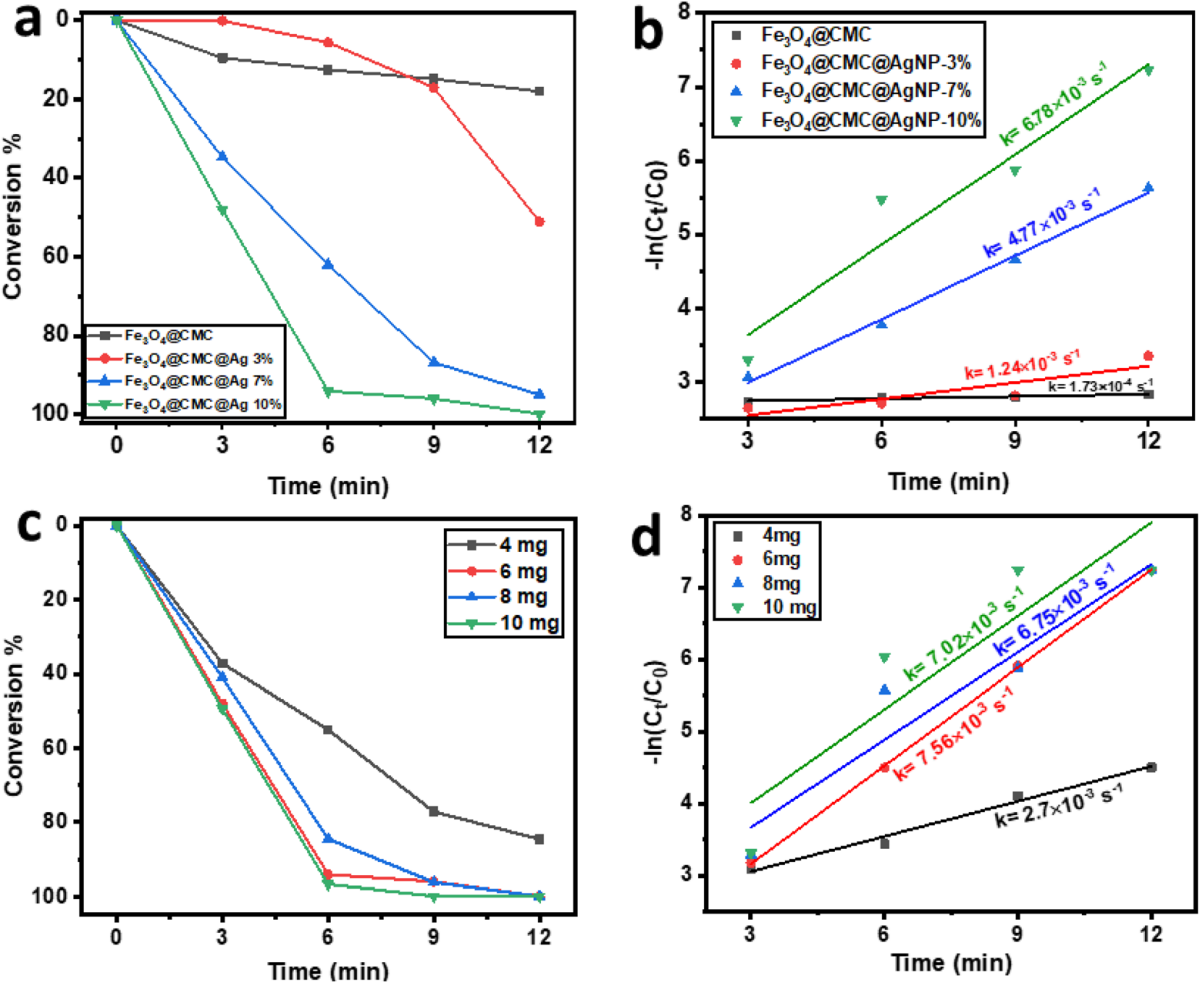
(a) Plots of conversion *vs.* reaction time and (b) plots of −ln(*C*_*t*_/*C*_0_) *vs.* reaction time of 4-NP with Fe_3_O_4_/CMC, Fe_3_O_4_/CMC-Ag-3%, Fe_3_O_4_/CMC-Ag-7% and Fe_3_O_4_/CMC-Ag-10%. (c) Plots of conversion *vs.* reaction time and (d) plots of −ln(*C*_*t*_/*C*_0_) *vs.* reaction time of 4-NP with Fe_3_O_4_/CMC, Fe_3_O_4_/CMC-Ag-3%, Fe_3_O_4_/CMC-Ag-7% and Fe_3_O_4_/CMC-Ag-10% with different dosages.

To evaluate the reduction process, the reaction mixture was stirred at room temperature without a catalyst, resulting in no change in the intensity of the 400 nm nitrophenolate peak after 60 min due to electrostatic repulsion between the compounds. The impact of a catalyst was examined using pristine Fe_3_O_4_/CMC and Fe_3_O_4_/CMC-AgNPs containing 3%, 7%, and 10% Ag NPs. The catalyst without Ag NPs exhibited minimal activity, converting only 17% of the 4-NP compound in 12 min. This reduction was evidenced by a decrease in the 400 nm absorption peak for 4-NP and an increase at 300 nm for 4-AP. Subsequently, the reduction process was studied with Fe_3_O_4_/CMC-AgNP catalysts, and the kinetics of each reaction was assessed (Fig. 4a and b). The findings indicated that increasing the percentage of composite Ag NPs enhances the reaction kinetics, thereby accelerating the reduction process, attributed to the superior electron-donating capability of the metal nanoparticles on the catalyst's surface. Following an analysis of the reaction kinetics, it was determined that the Fe_3_O_4_/CMC-AgNP-10% nanocatalyst exhibited the highest reduction efficiency. Consequently, the optimization of catalyst dosage was investigated as illustrated in Fig. 4c and d, where the amounts of 4, 6, 8, and 10 mg of Fe_3_O_4_/CMC-AgNPs-10% were tested against a constant concentration of 4-NP (0.072 mM) and NaBH_4_ (10 mg). The induction period for the 4, 6, and 8 mg catalyst dosages was less than 12 min, concluding the regeneration process. However, when the catalyst dosage was increased to 10 mg, this period decreased to 9 min, yet this was accompanied by a decrease in the reaction kinetics, yielding a rate constant of 7.02 × 10^−3^ s^−1^, which is lower than the kinetic value of 7.56 × 10^−3^ s^−1^ observed with the 6 mg catalyst. This decline in reaction kinetics may be attributed to the saturation of the catalyst's active sites by equal amounts of BH_4_^−^ and 4-NP. To further assess the catalytic activity of the synthesized catalyst in comparison with other catalysts, the turnover frequency (TOF, mmol mg^−1^ s^−1^) was calculated using eqn (3):3
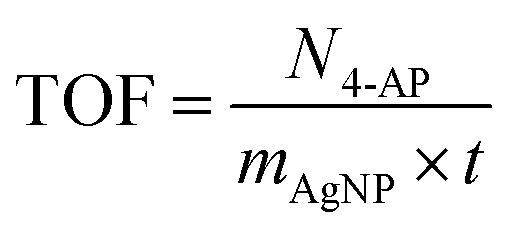


In this equation, *N*_4-NP_ is the mmol of 4-NP reduced, *m*_AgNP_ (mg) is the immobilized amount of AgNP on the Fe_3_O_4_/CMC substrate, and *t* is the reaction time (*s*). In order to assess the synthesized catalyst in relation to other catalysts, the turnover frequency (TOF, mmol mg^−1^ s^−1^) was calculated using eqn (3) to provide a more thorough evaluation of its catalytic performance. This calculation included determining the amount of 8.92% silver nanoparticles in 6 mg of the catalyst and measuring 7.2 × 10^−3^ mmol of 4-NP, which is equal to 99.9% conversion to 4-AP, along with the duration of the reaction. The TOF value obtained from these calculations is 2.24 × 10^−4^ s^−1^.

**Fig. 5:**
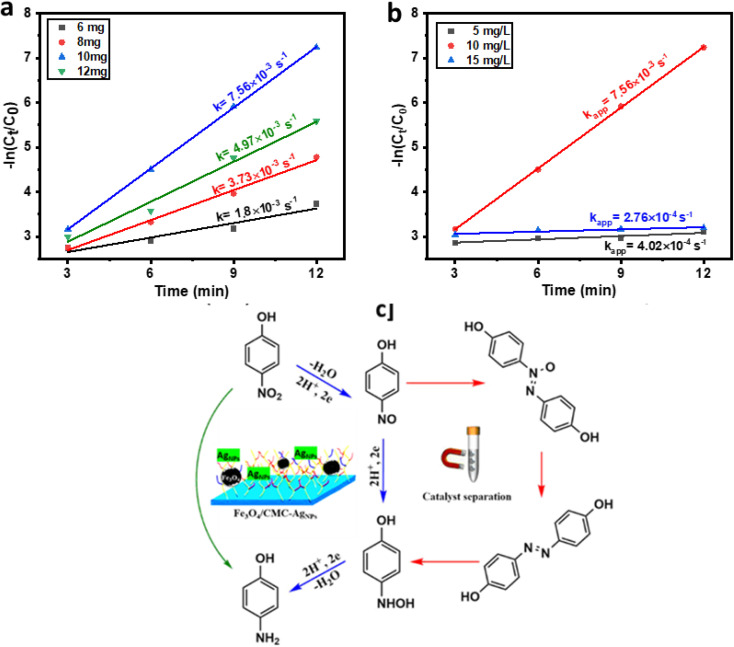
(a) Plots of −ln (*C*_*t*_/*C*_0_) *vs.* reaction time of NaBH_4_ optimization. (b) plots of −ln (*C*_*t*_/*C*_0_) *vs.* reaction time of concentration optimization of 4-NP. (c) Plausible mechanism for 4-NP reduction to 4-AP.

The catalytic reduction mechanism of 4-NP in the presence of sodium borohydride (NaBH_4_) can be elucidated using the Langmuir–Hinshelwood model. Initially, BH_4_^−^ ions adsorb onto the surface of the nanocatalyst, resulting in the formation of a metal hydride. As illustrated in the accompanying diagram, it is evident that, while maintaining a constant concentration of 4-nitrophenol, the reaction kinetics enhance with an increase in the amount of NaBH_4_ utilized as an auxiliary regenerator. However, when the concentration of NaBH_4_ becomes excessively high, the active sites on the catalyst surface become saturated with BH_4_^−^ ions, thereby impeding the adsorption of nitrophenolate ions onto the catalyst's active surface. Notably, the addition of 10 mg of NaBH_4_ yielded the highest reduction rate for the 4-NP compound, which is deemed optimal (Fig. 5a). Concurrently, as hydrogen species are absorbed, nitrophenolate ions also adhere to the catalyst surface through π–π stacking interactions. In the diagram presented in Fig. 5b, it is observed that the reaction kinetics significantly enhance as the concentration of 4-NP increases from 5 to 10 mg L^−1^. However, a notable decline in kinetics occurs with a further increase in concentration from 10 to 15 mg L^−1^. This phenomenon clearly indicates that the active site of the catalyst is becoming saturated by nitrophenolate ions, thereby hindering the interaction of BH_4_^−^ ions with the catalyst surface. Subsequently, an electron is transferred from the catalyst surface to 4-NP, leading to the reduction of the nitro group to a nitroso group, followed by conversion to an amino hydroxyl group and ultimately to the amino group. Finally, the resultant 4-AP is desorbed from the catalyst surface, allowing the catalyst to be ready for subsequent cycles (Fig. 5c).

**Fig. 6:**
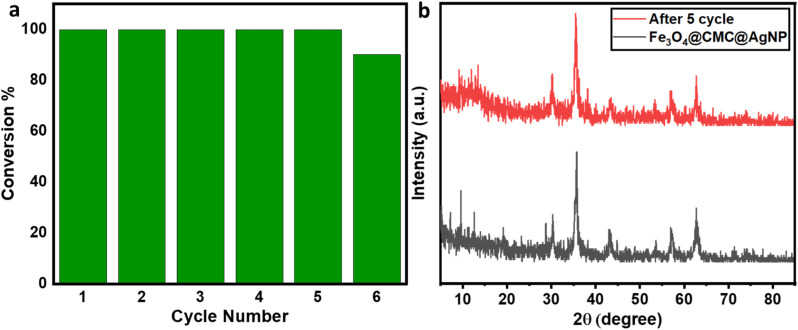
(a) The reusability cycles of Fe_3_O_4_/CMC-Ag-10%. (b) XRD patterns of Fe_3_O_4_/CMC-Ag-10% before and after 5 cycles.

In order to evaluate the resistance, reusability, and strength of nanoparticles on the nanocatalyst substrate, the nanoparticles were employed repeatedly in the reduction process of 4-nitrophenol. The stability of the catalyst was preserved even after five consecutive applications, and it was easily separable using a magnet for each instance (Fig. 6a). After each use, the catalyst underwent washing with distilled water, followed by ethanol, and was subsequently dried to ensure its readiness for the next cycle. The crystal structure stability was assessed through X-ray diffraction (XRD) analysis (Fig. 6b). The results obtained demonstrate the exceptional catalytic efficiency of Fe_3_O_4_/CMC-AgNPs.

## 4 Conclusion

In conclusion, carboxymethyl cellulose (CMC) biopolymer was synthesized to form a hybrid structure on Fe_3_O_4_ magnetic nanoparticles. This enhancement was aimed at increasing the surface area and preventing nanoparticle agglomeration. Subsequently, silver nanoparticles were incorporated onto this structure to facilitate the reduction of 4-nitrophenol. The resulting Fe_3_O_4_/CMC-AgNP nanocomposite exhibited significant magnetic properties, as indicated by its VSM measurements, and the amount of silver deposited on the substrate was confirmed through energy-dispersive X-ray (EDX) analysis following multiple washes. The optimal quantities of catalyst, sodium borohydride (NaBH_4_), and 4-nitrophenol concentration were identified, and the reaction mechanism was elucidated. Furthermore, the catalyst exhibited commendable stability over five consecutive cycles. Collectively, the Fe_3_O_4_/CMC-AgNP nanocomposite has proven to be an effective catalyst for the catalytic reduction of organic pollutants in wastewater treatment, yielding promising results.

## Data availability

All data generated or analysed during this study are included in this published article.

## Conflicts of interest

There are no conflicts to declare.
